# Maternal Obesity Related to High Fat Diet Induces Placenta Remodeling and Gut Microbiome Shaping That Are Responsible for Fetal Liver Lipid Dysmetabolism

**DOI:** 10.3389/fnut.2021.736944

**Published:** 2021-12-15

**Authors:** Ying-Wen Wang, Hong-Ren Yu, Mao-Meng Tiao, You-Lin Tain, I-Chun Lin, Jiunn-Ming Sheen, Yu-Ju Lin, Kow-Aung Chang, Chih-Cheng Chen, Ching-Chou Tsai, Li-Tung Huang

**Affiliations:** ^1^Department of Obstetrics and Gynecology, Chang Gung Memorial Hospital-Kaohsiung Medical Center, Kaohsiung, Taiwan; ^2^Department of Pediatrics, Chang Gung Memorial Hospital-Kaohsiung Medical Center, Graduate Institute of Clinical Medical Science, Chang Gung University College of Medicine, Kaohsiung, Taiwan; ^3^Department of Anesthesiology, Kaohsiung Chang Gung Memorial Hospital, Chang Gung University, College of Medicine, Kaohsiung, Taiwan

**Keywords:** maternal, high-fat diet, placenta, microbiome, oxidative stress, lipid metabolism, DOHaD

## Abstract

**Background:** Maternal obesity *in utero* may affect fetal development and cause metabolic problems during childhood and even adulthood. Diet-induced maternal obesity can impair gut barrier integrity and change the gut microbiome, which may contribute to adverse placental adaptations and increase the obesity risk in offspring. However, the mechanism through which maternal obesity causes offspring metabolic disorder must be identified.

**Methods:** Eight-week-old female rats received a control diet or high-fat (HF) diet for 11 weeks before conception and during gestation. The placentas were collected on gestational day 21 before offspring delivery. Placental tissues, gut microbiome, and short-chain fatty acids of dams and fetal liver tissues were studied.

**Results:** Maternal HF diet and obesity altered the placental structure and metabolism-related transcriptome and decreased G protein–coupled receptor 43 expression. HF diet and obesity also changed the gut microbiome composition and serum propionate level of dams. The fetal liver exhibited steatosis, enhanced oxidative stress, and increased expression of acetyl-CoA carboxylase 1 and lipoprotein lipase with changes in maternal HF diet and obesity.

**Conclusions:** Maternal HF diet and obesity shape gut microbiota and remodel the placenta of dams, resulting in lipid dysmetabolism of the fetal liver, which may ultimately contribute to the programming of offspring obesity.

## Graphical Abstract

Scheme of fetal steatosis induced by a maternal high-fat diet. High-fat (HF) diet intake during pregnancy remodels placenta and shapes the composition of the maternal gut microbiota, resulting in increased oxidative stress and lipid metabolism disruption of the fetal liver, which may ultimately lead to the programming of offspring obesity. 
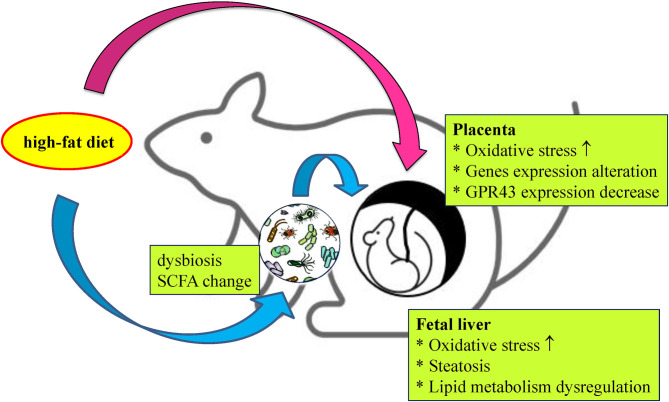

## Introduction

Obesity has rapidly increased in prevalence to become a major health problem (1). Maternal obesity can increase the risk of pregnancy complications, including pre-eclampsia, gestational diabetes mellitus, cesarean delivery, and preterm birth (2). In addition, maternal obesity and *in utero* maternal high-fat (HF) diet also affect fetal development and cause metabolic problems during childhood, adolescence, and adulthood (3). The Developmental Origins of Health and Disease (DOHaD) concept emphasizes the role of prenatal or perinatal exposure to environmental factors in determining the development of human diseases during adulthood (4). The fetus physically changes in response to environmental stress, which can increase disease risk. This programming effect is dependent on the nature and time of exposure. The DOHaD concept has been supported by numerous epidemiological and animal studies. Studies have revealed that early undernutrition impairs fetal growth and immune function in early life and increases the incidence of type-2 diabetes mellitus, cardiovascular disease, kidney disease, obesity, hypertension, osteoporosis, and metabolic syndrome later in life (4, 5). In addition to undernutrition, environmental factors such as maternal stress, infection, obesity, malnutrition, drug use, and cigarette smoke within indicated critical windows of growth and development are also associated with an increased risk of adult metabolic disease (6). Efforts to prevent non-communicable diseases have focused on adult factors; the DOHaD recommends the prioritization of optimizing nutrition early in life and reducing exposure to toxic environmental chemicals (7).

The placenta is the pivotal interface between the mother and developing embryo/fetus and plays multifunctional roles in fetal growth. Placental functions include attaching the developing fetus to the uterine wall, intervening in maternal immune tolerance, producing hormones, absorbing nutrients, removing waste, exchanging gas, and preventing the entry of chemical hazardous substances through the maternal–fetal blood supply during fetal development (8). Placental dysfunction and damage can adversely affect fetal development. The maternal nutrient supply passes to the fetus through the placenta, and the structure and function of the placenta change in response to the maternal nutrient supply. These changes affect the supply of nutrition and oxygen to and the distribution of hormones in the fetus. Evidence has indicated that obesity in pregnant women can disrupt placental function and cause adverse pathology in the perinatal period. Structural and molecular changes in the placenta of obese dams have been reported. Kretschmer et al. reported a decreased volume fraction of the labyrinth zone with excessive lipid accumulation, impaired trophoblast differentiation, and the downregulation of cell adhesion molecules in obese maternal mice exposed to an HF diet (9). Qiao et al. reported that a maternal HF diet induces lipoprotein lipase (LPL) expression in trophoblasts of placenta accompanied by Sirtuin 1 reduction and peroxisome proliferator–activated receptor gamma (PPARγ) enhancement (10). Other mechanisms of placental damage with maternal HF diet include oxidative damage, endoplasmic reticulum stress, and changes in nutrition sensing and nutrient transport (11–14). In a previous study, we demonstrated an association between placental renin–angiotensin system (RAS) activation and HF diet–induced fetal growth restriction (15). Although we have a preliminary understanding of how maternal obesity affects the placenta, more research is required to understand how maternal obesity remodels the placenta and thus programs offspring obesity and even worsens the development of non-communicable diseases in adulthood.

A well-balanced gut microbiome is crucial for homeostasis. Intestinal microbiota are closely related to metabolic diseases, such as obesity, diabetes, and hypertension (16). Maternal HF diet changes the gut microbiome of offspring, and gut microbiome shifts are closely associated with the metabolic parameters of such offspring (17). Because the fetal gut is colonized mainly by microbiota from the vaginal and fecal microbiome of the mother during delivery, the transfer of the maternal gut microbiome may play a crucial role in offspring metabolism. Short-chain fatty acids (SCFAs), composed of less than six carbon atoms, are mainly derived through the fermentation of indigestible dietary fiber by the intestinal microbiome. SCFAs, in addition to supplying energy, can modulate metabolism and exert anti-inflammatory, antitumorigenic, and antimicrobial effects (18, 19). SCFAs are mediated through the free fatty acid receptor (FFAR). GPR41 and GPR43, also known as “FFAR3” and “FFAR2,” respectively, are the most crucial receptors for SCFAs (19).

An HF diet during pregnancy and lactation has long-term consequences on the development of an offspring's gut microbiome (17). Diet-induced maternal obesity decreases the levels of maternal intestinal SCFAs and their receptors, diminishes the integrity of the gut barrier, and changes the gut microbiome, which may contribute to adverse placental adaptations and therefore increase the obesity risk in offspring (13). However, the signaling molecules that are relevant to placental adaptation and dysmetabolism in relation to maternal HF diet have not been clearly elucidated. To investigate the relationship between fetal programming and HF diet and obesity, next-generation sequencing (NGS) analysis of placentas was performed to determine the transcriptome expression after HF diet treatment. Placental adaptation and diet-induced maternal obesity change the gut microbiome and related metabolic pathways, thereby increasing the risk of obesity in offspring.

## Materials and Methods

### Study Animals and Experimental Design

The experimental animal protocol was approved by the Institutional Animal Care and Use Committee of Chang Gung Memorial Hospital (approval number: 2019053001). Twelve virgin female Sprague–Dawley rats aged 7 weeks were purchased from BioLASCO (BioLASCO Taiwan, Taipei, Taiwan). The rats were housed in a light-, temperature-, and humidity-controlled environment (12-h light–dark cycle, 22°C, and 55% humidity). Food and sterile tap water were available *ad libitum* (20). After a 1-week adaption to the experimental environment (maternal age: 8 weeks), the rats were weight matched and assigned to receive either a regular control diet (59.7% carbohydrates, 27.5% protein, 12.6% fat by energy, 3.25 kcal/gm; Fwusow Industry, Taichung, Taiwan) (CC group) or an HF diet (D12331, Research Diets, New Brunswick, NJ, USA; 58% fat [hydrogenated coconut oil], 16.4% protein plus high sucrose [25% carbohydrate] by energy, 5.56 kcal/gm) (HF group) (*n* = 6 per group). The rats were fed the assigned diet for 8 weeks (maternal age: 16 weeks) and maintained in an environment conducive to mating for 3 days. The assigned diet was continued until the day of sacrifice. Mating day 1 was considered gestational day 1. The beginning of the gestation was confirmed by checking the vaginal plug. Because rats deliver their pups on day 22 or 23 of the gestation period (21), the maternal rats were sacrificed on gestational day 21 after 8 h of fasting (maternal age: 19 weeks). The offspring subjects of each group came from different litters.

### Specimen Collection

The rats were sacrificed through anesthetization with a 1:1 mixture of Zoletil (25 mg/kg) (tiletamine-zolazepam, Virbac; Carros Cedex, France) and Rompun (23.32 mg xylazine hydrochloride, Bayer, Korea) administered through intramuscular injection. Heparinized blood samples were collected through cardiocentesis (22, 23). The placenta of the dams and fetal liver were collected through a cesarean section of the rats. A part of the placenta was fixed in 10% formalin in a neutral buffered solution for histological analysis, and the remainder was frozen in liquid nitrogen and stored at −80°C for NGS and quantitative polymerase chain reaction (qPCR) analysis (*n* = 6 per group). Furthermore, the retroperitoneal adipose depot was sampled through a procedure that was identical to that of our previous study (23).

### Body Weight and Blood Pressure Measurement

The body weights of the rats were measured weekly from 7 weeks of age until the day of sacrifice. The blood pressure (BP) of the rats was measured at 5 days before sacrifice by using the indirect tail-cuff method (BP-2000, Visitech Systems, Apex, NC, USA) as previously described (23).

### Intraperitoneal Glucose Tolerance Test

Blood sugar levels were measured using the intraperitoneal glucose tolerance test (IPGTT) after an 8-week dietary manipulation. On the day of the IPGTT, the rats fasted for 8 h and hyperglycemia was induced through the injection of 50% glucose (2 g/kg body weight). Serum glucose levels were measured in blood from the tail vein using a glucometer (Accu-Chek, Roche, Germany) at five time points: before injection and at 15, 30, 60, and 120 min after injection. The IPGTT's integrated area under the curve (AUC) was calculated using the trapezoidal method.

### Biochemical Analysis

Some plasma metabolic parameters of maternal rat blood samples were analyzed, including glutamic-oxalocetic transaminase (GOT) level, glutamic-pyruvic transaminase (GPT) level, total cholesterol (T-chol), and leptin. Serum GOT, GPT, and T-chol levels were evaluated using an automatic biochemical analyzer (Fuji Dry-chem 4400i; Fujifilm, Tokyo, Japan). Serum leptin levels were measured using an enzyme-linked immunosorbent assay kit (Abcam, Cambridge, MA, USA) (*n* = 6 per group).

### Histological Analysis of the Placenta and Fetal Liver

Formalin-fixed tissues were cut into 3-μm sections by using a Leica RM2255 microtome (Leica Biosystems, Concord, ON, Canada). These sections were first stained with hematoxylin and eosin (H&E) and then scanned with a 3DHISTECH Panoramic SCAN slide scanner. The scanned image was further analyzed using Panoramic Viewer software. The placental composition was analyzed using ImageJ at 1.5 × magnification. A major product of DNA oxidation is 8-hydroxy-2-deoxyguanosine (8-OHdG), which is often used as a biomarker for oxidative stress (24). The oxidative stresses of both the placenta and fetal liver were determined using 8-OhdG (25). The tissue sections were transferred to polylysine-coated slides and incubated with primary anti-8-OhdG antibody (Santa Cruz Biotechnology, CA, USA) for 60 min at room temperature. After rinsing was conducted, the sections were incubated with secondary antibody for 30 min at room temperature and thereafter incubated with Avidin and biotinylated horseradish peroxidase H. The horseradish peroxidase converted the diaminobenzidine tetrahydrochloride substrate into an insoluble dark brown precipitate. To investigate the effects of a maternal HF diet on the fatty liver and the mechanism by which this developmental priming is mediated, fetal livers were also indicated for study.

### RNA Isolation, Library Preparation, and NGS and Analysis

The method for RNA isolation was identical to that described in a previous study (26). In brief, the total RNA of placental tissue was extracted using Trizol Reagent (Invitrogen, USA) according to the manufacturer's instructions. After quantification was conducted using an ND-1000 spectrophotometer (Nanodrop Technology, USA) and Bioanalyzer 2100 (Agilent Technology, USA), the Select Strand-Specific RNA Library Preparation Kit was used for library construction prior to the use of AMPure XP beads (Beckman Coulter, USA) for size selection. Illumina's sequencing-by-synthesis technology (Illumina, USA) was used to determine the RNA sequence. Sequencing data (FASTQ reads) were generated using Welgene Biotech's pipeline based on Illumina's base calling program bcl2fastq v2.20. After the removal of low base quality data, of polymerase chain reaction (PCR) primers, and of other artifacts, quality trimming was conducted using Trimmomatic version 0.32. Transcriptome alignment was performed using HISAT2. Reads per kilobase of exon per million mapped reads were quantified to determine gene expression. The Cuffdiff tool from the cuf?inks package was run to calculate expression changes and associated q values (*P* values adjusted for the false discovery rate) for each gene between the control and HF groups. Differentially expressed genes of each experiment design were subjected to an enrichment test for a functional assay by using clusterProfiler 3.5. Records in the Gene Ontology database and Kyoto Encyclopedia of Genes and Genomes (KEGG) were matched with the data using NIH DAVID Bioinformatics Resources 6.7 to determine candidate genes and pathways.

### Quantitative Real-Time PCR Analysis

To validate the transcriptome expression of the placenta and evaluate the lipid metabolism of the fetal liver, the messenger RNA (mRNA) expressions of the placenta and fetal liver were analyzed through qPCR. Placentas were collected from the study rats. RNA extraction and qPCR protocols were performed per the method of previous studies (23, 27); the primer sequences of mRNA are presented in Supplementary Table 1. In addition, 18S ribosomal RNA and glyceraldehyde 3-phosphate dehydrogenase (GAPDH) were used as housekeeping genes for the placenta and fetal liver tissue, respectively. To evaluate the relative quantification, we adopted the comparative threshold cycle method (23, 27).

### Microbial Analysis

#### DNA Extraction and PCR Amplification

Fecal samples for every rat were collected in individual 5-mL Eppendorf Tubes 1 week before they were sacrificed. The samples were snap-frozen with liquid nitrogen and stored at −80 °C until analysis. Microbial DNA in the stool samples was extracted using a EZNA Soil DNA Kit (Omega Bio-tek). The V3–V4 region of the bacterial 16S ribosomal RNA (rRNA) gene was amplified using PCR with primers 338F (5′-ACT CCT ACG GGA GGC AGC A-3′) and 806R (5′-GGA CTA CHV GGG TWT CTA AT-3′). The barcode was an N-base sequence (N represents a 6–8 nucleotide), which was unique to each sample. The PCR protocol was identical to that used in our previous study (17).

#### Sequencing

Amplicons were purified from 2% agarose gels by using an AxyPrep DNA Gel Extraction Kit (Axygen Biosciences). Purified amplicons were quantified using QuantiFluor-ST (Promega). Processed amplicons were pooled in equimolar and paired-end sequences (2 × 300) and analyzed on an Illumina MiSeq platform.

#### Bioinformatic Analysis

The lowest sequencing reads were selected from each sample with the assistance of the pseudorandom generator, and samples were compared with respect to community composition and structure. Raw fastq files were analyzed using QIIME (version 1.17) according to the following three criteria. First, 300-bp reads were truncated at any site receiving an average quality score of b20 over a 10-bp sliding window and reads <50 bp were discarded. Second, barcodes were to be exactly matched, where primers with a nucleotide mismatch and reads containing ambiguous characters were removed. Third, only sequences overlapping for > 10 bp were assembled according to their overlapped sequence; reads that could not be assembled were discarded. Operational taxonomic units were clustered with a 97% similarity cutoff using UPARSE (version 7.1 http://drive5.com/uparse/), and chimeric sequences were identified and removed using UCHIME. The phylogenetic affiliation of each 16S rRNA gene sequence was analyzed using the RDP Classifier (http://rdp.cme.msu.edu/) against the Silva (SSU115) 16S rRNA database at a confidence threshold of 70%. To determine whether HF diet exposure creates a similar gut microbiota pattern, we evaluated the Firmicutes to Bacteroidetes (F/B) ratio.

### SCFA Analysis

To demonstrate the influence of the gut microbiota on a host, serum SCFA levels, the main fermentation product of the gut microbiota, were compared between the rats receiving an HF diet and those receiving the control diet. The plasma levels of acetic acid, propionic acid, and butyric acid were determined using gas chromatography (GC). Our previous study detailed the GC protocol (17). In brief, the 100-μL sample was mixed with 5 μL of 100-μM internal standard and 100 μL of propyl formate. After vertexing and centrifuging, the supernatant was injected for GC analysis (Shimazu QPlus 2010 gas chromatography with flame ionization detector [FID]). The injection volume was 2 μL, and the inlet and FID temperatures were 200 and 240°C, respectively.

### Statistics

Differences between the HF and CC groups were analyzed through the Mann–Whitney U test for dependent variables that are not normally distributed. Values are expressed as the mean ± standard error of the mean, and a *P* value of <0.05 was considered statistically significant. A repeated-measure analysis of variance model was used to determine the body weight (BW) difference and for the IPGTT test between the groups. The BW of the rats was measured weekly from 8 weeks of age until sacrifice. The IPGTT was performed 5 days before sacrifice. The interaction between group and time (G × T) was calculated for each variable. All statistical analyses were performed using SPSS 22.0 for Windows XP (SPSS, Chicago, IL, USA).

## Results

### HF Diet Causes Obesity and Alters the Metabolic Profile of Dams

**Figure 1:**
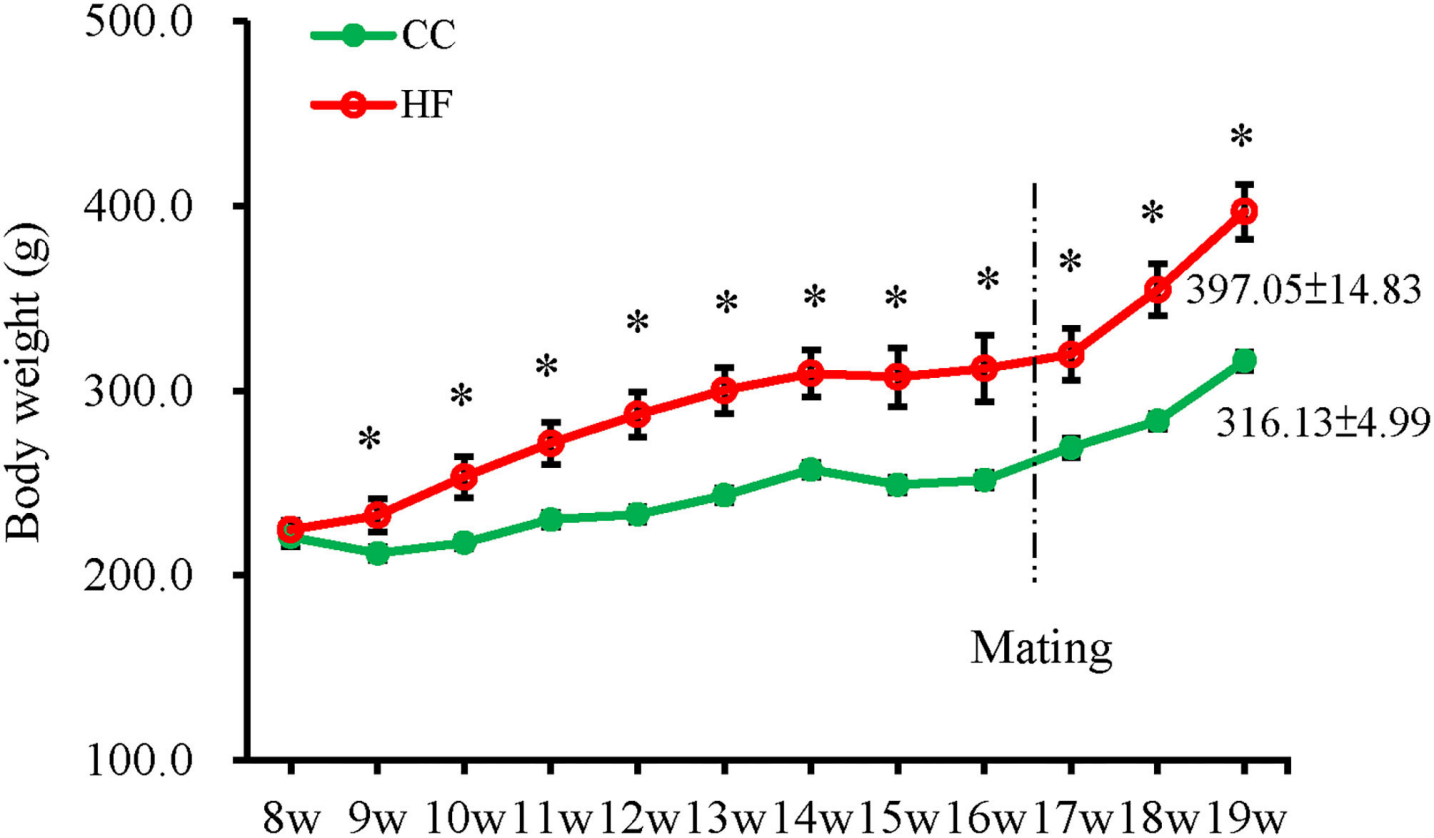
Change in body weights of dams after control diet (CC) or high-fat diet (HF). The results are presented as the mean ± standard error. A significant difference was observed after undergoing the diet for 1 week. A repeated-measure ANOVA model was used to test the difference in BW between the groups (*n* = 6 for each group). *P < 0.05.

**Figure 2:**
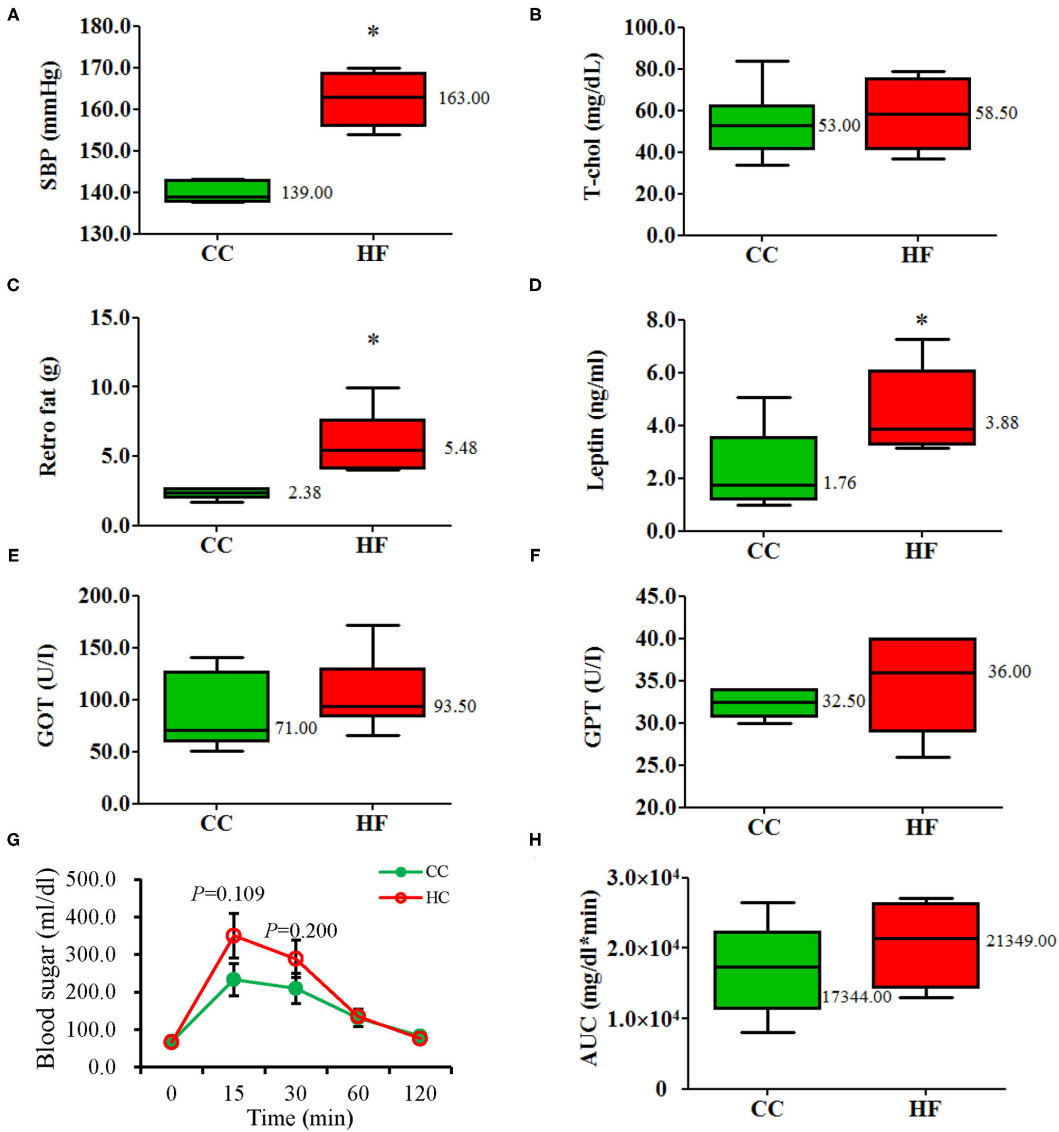
Metabolic profiles of the dams after 11 weeks of undergoing the diet (19-week-old). **(A)** SBP, **(B)** T-cho, **(C)** Retro fat, **(D)** Leptin, **(E)** GOT, **(F)** GPT, **(G)** blood sugar, **(H)** AUC. Compared with the control group (CC), the high-fat diet group (HF) had higher systolic blood pressure, greater retroperitoneal fat deposits, and higher plasma leptin levels. The integrated AUC values of the intraperitoneal glucose tolerance test were calculated using the trapezoidal method. All values are presented as mean ± standard error. A repeated-measure ANOVA model was used for the IPGTT test of the groups. Other parameters were analyzed using a Mann–Whitney *U* test (*n* = 6 for each group). *P < 0.05. The median was shown. SBP, systolic blood pressure; Retro, retroperitoneal, GOT, glutamic-oxalocetic transaminase; GPT, glutamic-pyruvic transaminase; T-chol, total cholesterol; AUC, glucose area under the curve.

The BWs of the rats were measured weekly from 8 weeks of age until the day of sacrifice (Figure 1). The HF group had a significantly higher BW than the CC group did after 1 week of exposure to the assigned diets (HF vs. CC; 232.42 ± 8.83 g vs. 211.83 ± 3.68 g; *P* = 0.037) until the end of the experiment (HF vs. CC; 397.06 ± 14.83 g vs. 316.13 ± 4.99 g; *P* = 0.004). Weight increased significantly in the HF group after 11 weeks of diet manipulation. Repeated measures indicated a main effect for time (*F*_11, 110_ = 155.635, *P* < 0.001) and group (*F*_1, 10_ = 15.837, *P* = 0.003) (G × T, *P* < 0.001). After 11 weeks of diet manipulation, the metabolic profiles of dams receiving an HF diet exhibited a significantly higher level of systolic BP, a larger volume of retroperitoneal fat, and higher serum leptin levels than those of dams receiving the control diet (Figure 2). GOT, GPT and T-chol values were similar between the CC and HF groups (Figures 2E,F). For IPGTT, the HF group exhibited a non-signficantly higher glucose level at 15 and 30 min than did the CC group (Figure 2G). The AUC was also slightly but non-significantly higher (the main effect of time [*F*_4, 40_ = 31.584, P < 0.001] and group [*F*_1, 10_ = 1.427, *P* = 0.260]; G × T, *P* < 0.001) (Figure 2H).

### Exposure to HF Diet Changes Placental Structure and Expression of Metabolism-Related Genes

#### Exposure to HF Diet Decreases the Thickness of the Placental Labyrinth Zone and Increases 8-OHdG Expression

**Figure 3:**
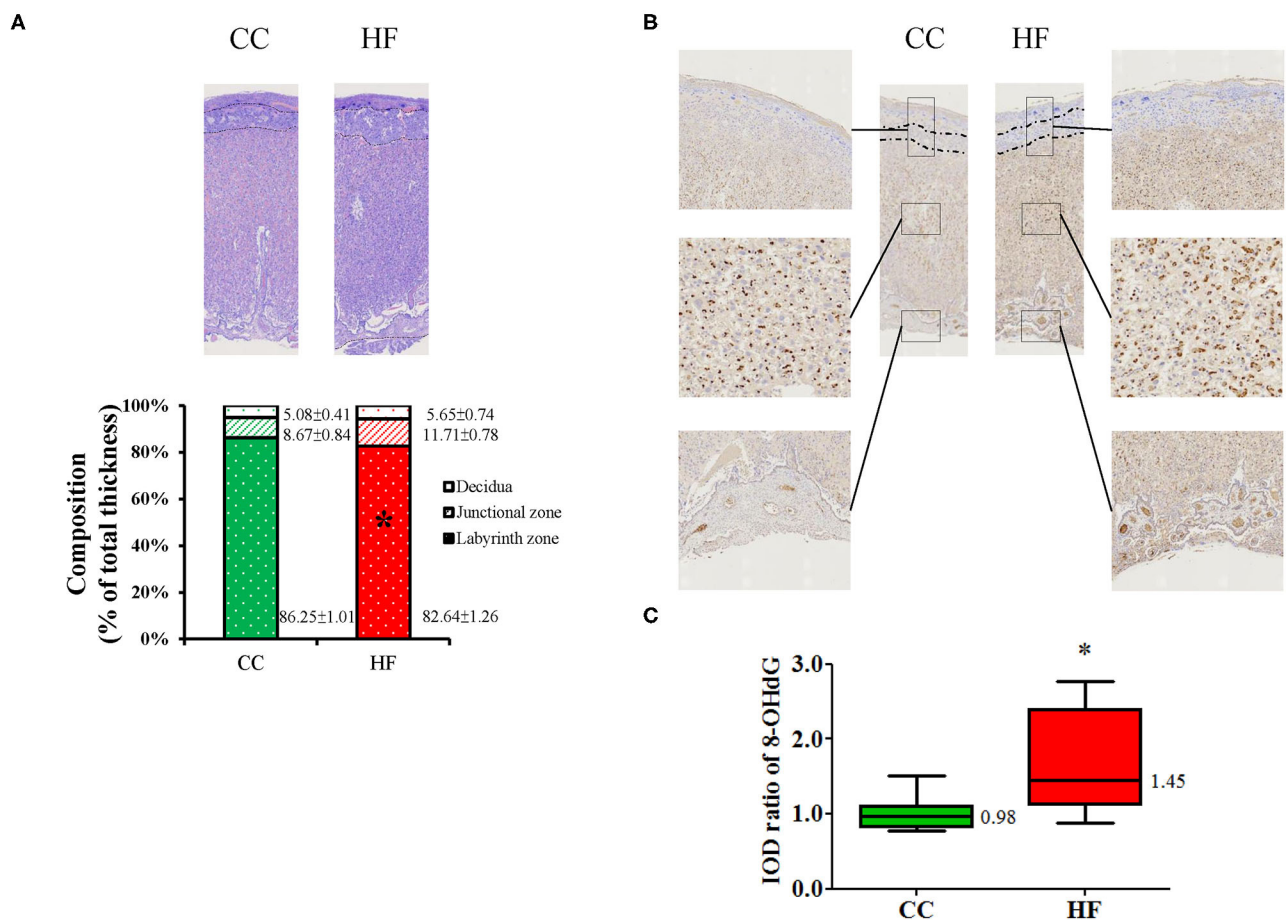
Histological changes in placenta after high-fat diet intake. **(A)** Histological appearance of placenta. Mean proportions of thicknesses of the decidua basalis, junctional zone, and labyrinth zone are indicated on a bar graph. **(B)** Oxidative stress was determined based on 8-hydroxy-2-deoxyguanosine (8-OHdG). **(C)** Relative quantitative analysis of 8-OHdG by IOD. Magnification of boxed area detailing the three layers of placenta and chorionic villi (V) (*n* = 6 for each group). *P < 0.05. The median was shown. CC, control group; HF, high-fat diet group; IOD, image optical density.

The placenta plays an essential role in fetal programming; therefore, the remodeling of the placenta by a maternal HF diet was studied. The CC and HF groups did not signifiacntly differ with respect to placental weight and litter characteristics, including number, size, and sex (Supplementary Table 2). Considering that the placenta contributes to direct nutrient exchange between fetal and maternal circulation, we analyzed placental adaptation due to the HF diet. The mature placenta is composed of three histologic zones, namely the maternal decidua on the outside, the junctional zone, and the inner labyrinth zone. A comparison of histologic zone thickness revealed that the labyrinth zone was significantly thinner in the HF group than in the CC group (HF vs. CC; 82.64% ± 1.26% vs. 86.25% ± 1.01%, *P* = 0.038, Figure 3A). Oxidative stress between the two groups was compared based on 8-OHdG staining. All three placental zones in the HF group exhibited greater 8-OHdG staining by an average of 1.67 ± 0.23 fold relative to the CC group (*P* = 0.015; Figure 3B).

### Maternal HF Diet Changes the Expression of Metabolism-Related Genes in Placenta

**Figure 4:**
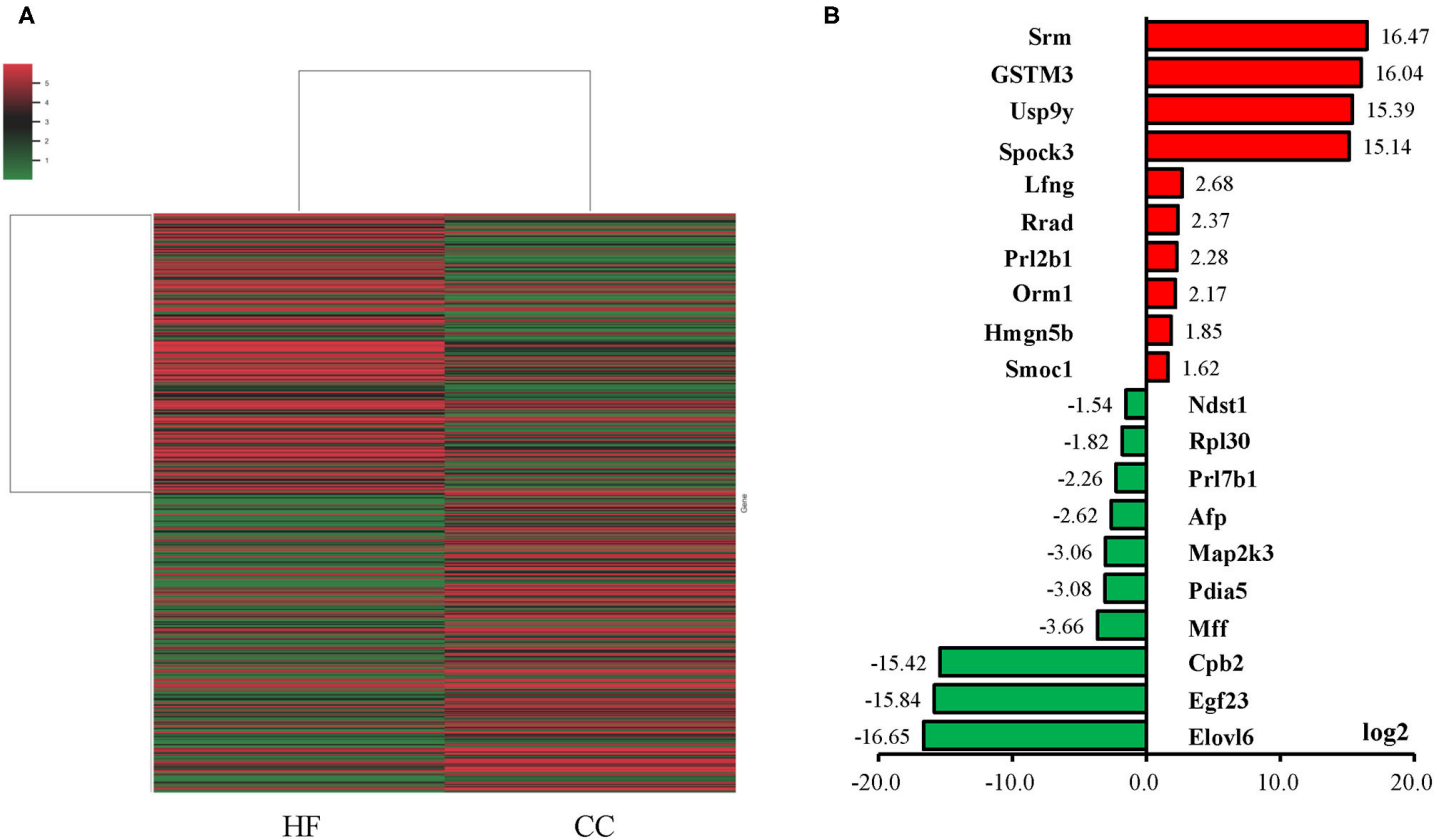
Gene expression of placental tissue in control diet (CC) group and high-fat diet (HF) group. **(A)** Heat map of mRNA expression determined using next-generation sequencing (NGS). The heat map lane represents the top 100 expressed mRNAs. Higher and lower intensities are indicated in red and green, respectively. Among the gene expressions with a 1.5-fold difference, the top 10 mRNAs expressed in the HF (red bar) and CC groups are indicated on the bar chart **(B)**. Expression of mRNAs is presented in terms of relative folds. Srm, spermidine synthase; GSTM3, glutathione S-transferase mu 3; Usp9y, ubiquitin specific peptidase 9, Y-linked; Spock3, SPARC/osteonectin, cwcv and kazal like domains proteoglycan 3; Lfng, LFNG O-fucosylpeptide 3-beta-N-acetylglucosaminyltransferase; Rrad, RRAD, Ras-related glycolysis inhibitor and calcium channel regulator; Prl2b1, prolactin family 2, subfamily b, member 1; Orm1, orosomucoid 1; Hmgn5b, high mobility group nucleosome binding domain 5B; Smoc1, SPARC related modular calcium binding 1; Ndst1, N-deacetylase and N-sulfotransferase 1; Rpl30, ribosomal protein L30; Prl7b1, prolactin family 7, subfamily b, member 1; AfP, alpha-fetoprotein; Map2k3, mitogen activated protein kinase kinase 3; Pdia5, protein disulfide isomerase family A, member 5; Mff, mitochondrial fission factor; Cpb2, carboxypeptidase B2; Egf23, fibroblast growth factor 23; Elovl6, ELOVL fatty acid elongase 6.

**Figure 5:**
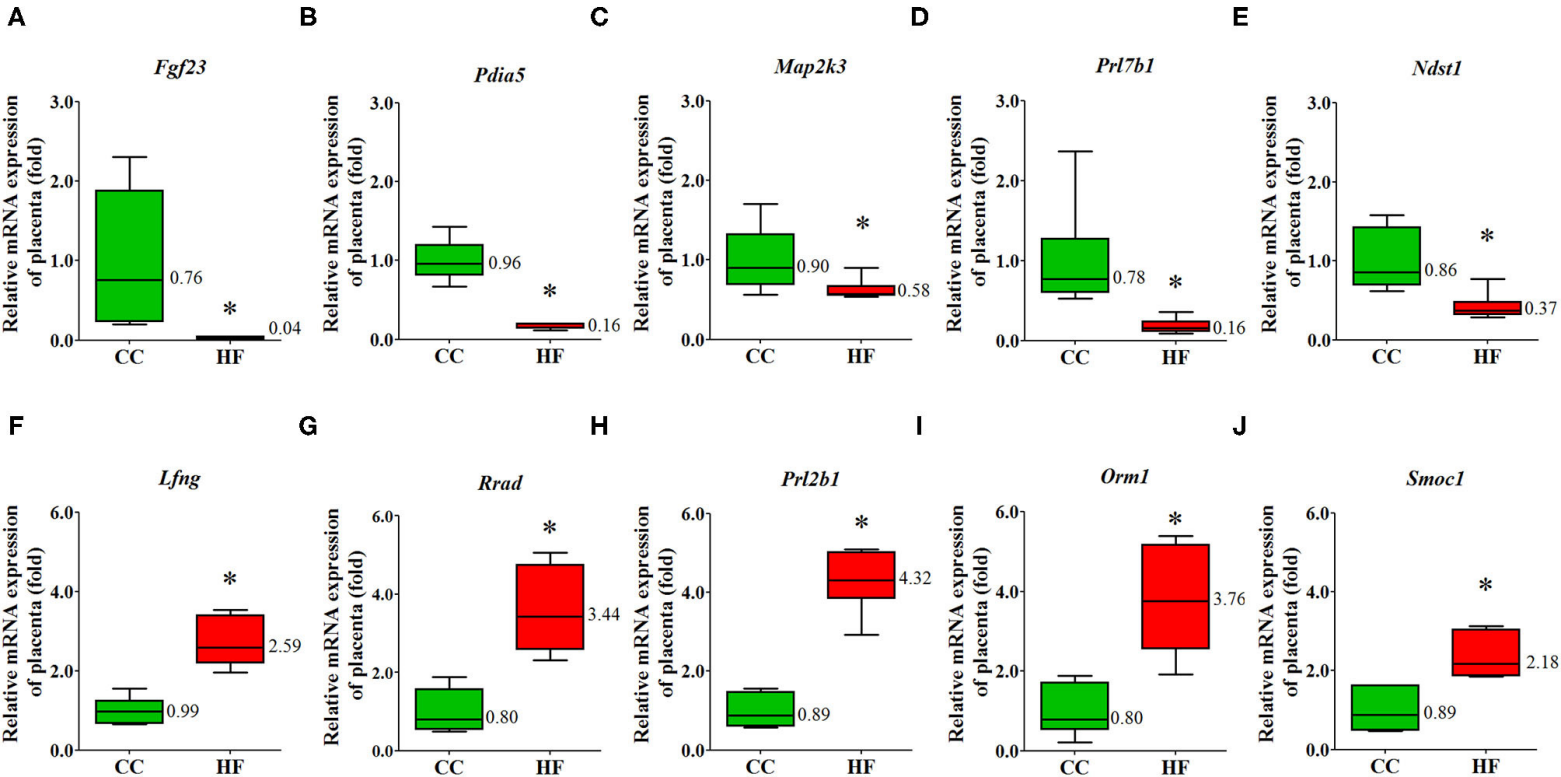
Validation of dysregulated placental mRNA. The expression of dysregulated genes was compared between rats exposed to a high-fat diet or a control diet. Reverse transcription–quantitative polymerase chain reaction analysis was conducted to validate the mRNA profiles of five mRNAs determined using next-generation sequencing (NGS). **(A)** *Fgf23*, **(B)** *Pdia5*, **(C)** *Map2k3*, **(D)** *Prl7b1* **(E)** *Ndst1*, **(F)** *Lfng*, **(G)** *Rrad*, **(H)** *Prl2b1*, **(I)** *Orm1*, **(J)** *Smoc1*. The results demonstrated that the expression patterns of these mRNAs are consistent with those determined by NGS. The housekeeping gene was 18S ribosomal RNA, and mRNA expression is presented in terms of relative fold. *P < 0.05 (*n* = 6 for each group). The median was shown. Fgf23, fibroblast growth factor 23; Pdia5, protein disulfide isomerase family A member 5; Map2k3, mitogen activated protein kinase 3; Prl7b1, prolactin-7B1; Ndst1, N-deacetylase and N-sulfotransferase 1; Lfng, LFNG O-fucosylpeptide 3-beta-N-acetylglucosaminyltransferase; Rrad, Ras-related glycolysis inhibitor and calcium channel regulator; Prl2b1, prolactin family 2, subfamily b, member 1; Orm1, orosomucoid 1; Smoc1, SPARC-related modular calcium binding 1.

An NGS analysis of placentas was performed to determine the transcriptome expression after an HF diet treatment. The total number of reads in the HF and CC groups were 84,978,988 and 100,611,252, with a mapping rate of 95.79 and 95.25%, respectively. The heat map revealed a substantial difference between the HF and CC groups in terms of mRNA expression (Figure 4A). Those mRNA with at least a 1.5-fold difference (*P* < 0.01) in expression between the placental tissues of the CC and HF groups were selected. The log2-fold change was used to classify the genes into upregulated and downregulated groups. We observed 26 and 27 upregulated (Supplementary Table 3) and downregulated (Supplementary Table 4) genes, respectively, in the HF group compared with in the CC group. Genes with the largest difference between the two groups are illustrated in Figure 4B. The 10 genes with the highest expression in the HF group compared with the CC group are indicated in red; they are Srm, GSTM3, Usp9y, Spock3, Lfng, Rrad, Prl2b1, Orm1, Hmgn5b, and Smoc1. The 10 genes with the lowest expression in the HF group compared with the CC group are shown in green; they are SPARC, Ndst1, Rpl30, Prl7b1, AfP, Map2k3, Pdia5, Mff, Cpb2, Egf23, and Elovl6. Among them, five upregulated and downregulated genes each were selected for qPCR validation. The results of the qPCR were compatible with those of the NGS (Figure 5). DAVID v6.7 was then used to identify functionally related gene groups. Functional annotation clustering revealed 19 significantly related KEGG pathways in the placental tissues of the HF group vs. the control group (Table 1). Ribosome was the most significant KEGG pathway. Other functional pathways altered by the maternal HF diet include cholesterol, arginine, and proline metabolism, oxidative phosphorylation, non-alcoholic fatty liver disease, and tight junction.

**Table 1:** KEGG pathways relevant to differently expressed genes in placental tissues of HF and CC groups.

**Description**	**Gene ratio**	**Bg ratio**	***P* value**	**q value**	**Gene ID**	**Count**
Ribosome	28/296	179/8567	0.000	0.000	LOC108352650/Rpl26/Rps27a/Rpl19/Rpl30/Rps24/ Rpl29/LOC100360522/Rps2/Rpl35/Rps12/Uba52/ Rps11/Rpl27/LOC687780/Rpl23a/Rps15/Rps18/ LOC102555453/LOC100362684/LOC100362027/ LOC100360841/Rps19/LOC103694404/LOC100359 687/Rpl7a/Rps27l/Rps10l1/	28
Complement and coagulation cascades	11/296	83/8567	0.000	0.015	Serpinc1/Fgb/Vtn/Cpb2/F2/Plat/Fga/Fgg/Serpina1/ C3/C7/	11
Cholesterol metabolism	7/296	51/8567	0.002	0.072	Apoh/Apob/Apoc2/Apoa1/Soat2/Apoa4/Lipc/	7
Transcriptional misregulation in cancer	15/296	184/8567	0.002	0.072	Cdkn1a/Lyl1/Nr4a3/Slc45a3/Ewsr1/Gadd45g/ Hhex/Csf1r/Plat/Pax8/LOC102549173/Hist3h3/ LOC103694865/Cebpb/Igfbp3/	15
Thermogenesis	18/296	243/8567	0.002	0.072	Prkg2/Cox4i2/Ndufa6/LOC100911615/Adcy4/ Ndufa13/Cox8a/Atp5mf/LOC100363268/Bmp8a/ COX2/Ndufa1/Ndufa11/Adcy9/LOC100361457/ Cox7b/Uqcr10/LOC103694876/	18
Arginine and proline metabolism	7/296	52/8567	0.002	0.072	Gatm/Aldh2/Maoa/Nos3/Srm/ LOC100912604/Nos2/	7
African trypanosomiasis	6/296	39/8567	0.002	0.072	Il12a/Hba1/Apoa1/Hba-a1/LOC103694857/Hbb/	6
p53 signaling pathway	8/296	74/8567	0.004	0.106	Sesn1/Cdkn1a/Zmat3/Gadd45g/Pmaip1/Ccnd1/ Ccnb1/Igfbp3/	8
Oxidative phosphorylation	12/296	143/8567	0.004	0.106	Cox4i2/Ndufa6/Lhpp/Ndufa13/Cox8a/Atp5mf/ LOC100363268/COX2/Ndufa1/Ndufa11/Cox7b/ Uqcr10/	12
Huntington disease	15/296	202/8567	0.004	0.106	Sod1/Cox4i2/Ndufa6/Hap1/Ap2s1/Ndufa13/Cox8a/ LOC100363268/COX2/Ndufa1/Bdnf/Ndufa11/Gpx1/ Cox7b/Uqcr10/	15
Platelet activation	11/296	129/8567	0.005	0.112	Prkg2/Pik3r6/Fgb/Nos3/NEWGENE_621351/Adcy4/ Rasgrp2/Fga/Fgg/Adcy9/LOC100361457/	11
Cardiac muscle contraction	8/296	81/8567	0.007	0.139	Cacng4/Cox4i2/Atp1b2/Cox8a/COX2/Tnnt2/ Cox7b/Uqcr10/	8
Apelin signaling pathway	11/296	141/8567	0.010	0.183	Gnb2/Apln/Pik3r6/Jag1/Aplnr/Nos3/Plat/Adcy4/ Ccnd1/Adcy9/Nos2/	11
Gap junction	8/296	88/8567	0.011	0.193	Prkg2/Tubb3/Adcy4/Tuba1c/Tubb4a/Tuba8/Adcy9/ Tuba1b/	8
Thyroid hormone synthesis	7/296	72/8567	0.012	0.197	Atp1b2/Ttr/Duox2/Adcy4/Pax8/Gpx1/Adcy9/	7
Tight junction	12/296	170/8567	0.015	0.229	Cldn15/LOC103694903/Rab13/Myh14/Ccnd1/ Tuba1c/Tiam1/Cldn5/Cttn/Tuba8/LOC100361457/ Tuba1b/	12
Parkinson disease	11/296	152/8567	0.016	0.236	Cox4i2/Ndufa6/Slc6a3/Ndufa13/Cox8a/ LOC100363268/COX2/Ndufa1/Ndufa11/Cox7b/ Uqcr10/	11
Phagosome	13/296	198/8567	0.020	0.272	Sec61g/Sec61b/Cyba/Tubb3/Tuba1c/RT1-A2/C3/Tubb4a/Tuba8/RT1- CE10/LOC108348139/LOC100361457/Tuba1b/	13
Non-alcoholic fatty liver disease (NAFLD)	11/296	159/8567	0.022	0.286	Mlxip/Cox4i2/Ndufa6/Ndufa13/Cox8a/LOC1003 63268/COX2/Ndufa1/Ndufa11/Cox7b/Uqcr10/	11

### HF Diet Shapes the Gut Microbiota and Decreases the Plasma Propionate Level of Dams

**Figure 6:**
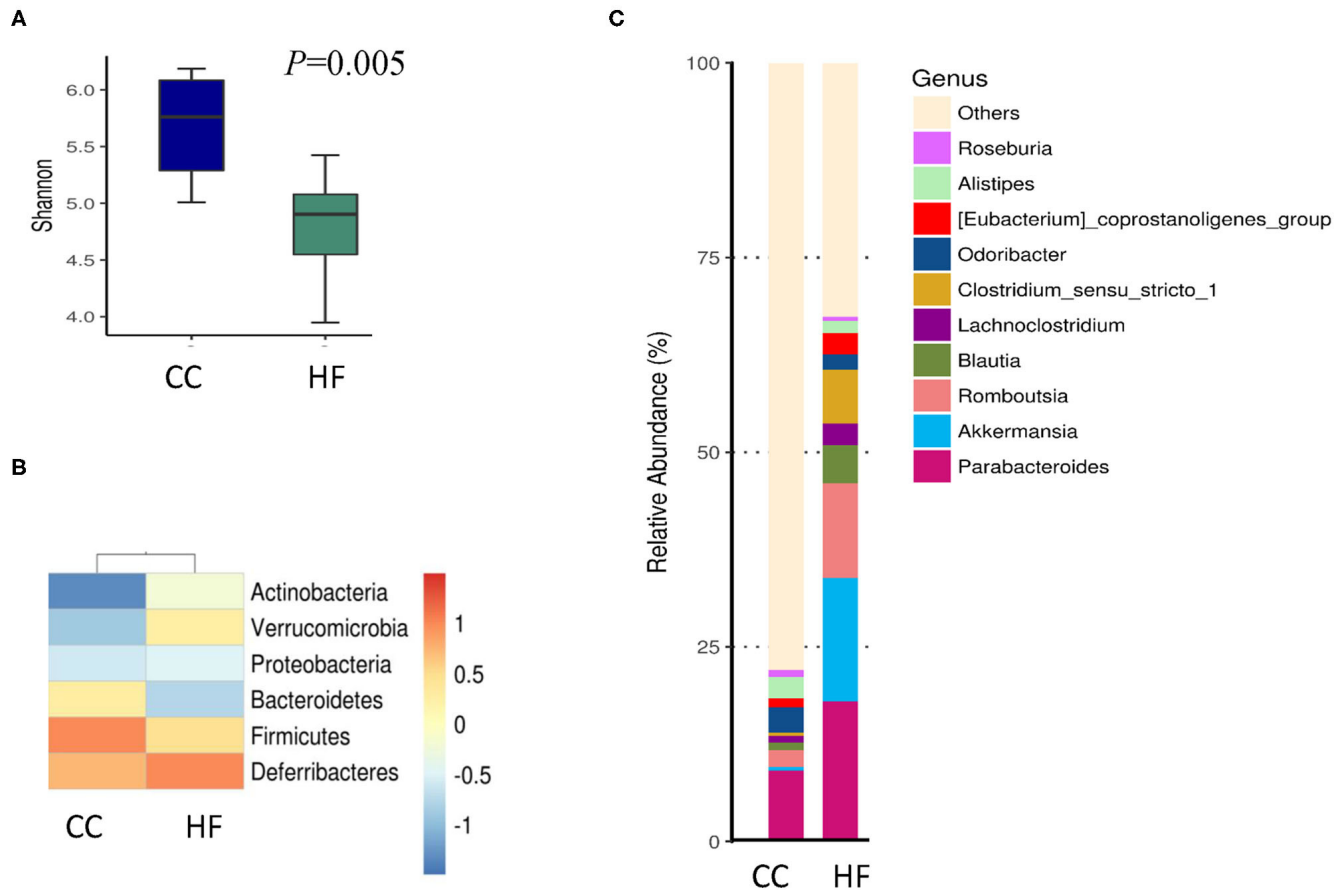
Effect of high-fat diet on composition of gut microbiota in dams. **(A)** Lower alpha diversity in high-fat diet (HF) rats as compared with control diet (CC) rats. *P < 0.05. **(B)** Phylum and **(C)** genus classification of gut microbiota from dams on CC or HF. Column plot indicating genus class with a coverage of >95% (*n* = 6 for each group). The most abundant spectra are listed.

To illustrate how maternal HF diet exposure changes the gut microbiome and then influences the metabolic parameters of the offspring, we determined the proportions of 16S rDNA reads assigned to each phylum for dams. The HF group exhibited a lower level of alpha diversity than did the CC group in terms of the Shannon index (Figure 6A). The beta diversity determined through a principal coordinate analysis indicated no significant difference between both groups (data not shown). The CC and HF groups exhibited distinct patterns both at the phylum (Figure 6B) and genus (Figure 6C) levels.

**Figure 7:**
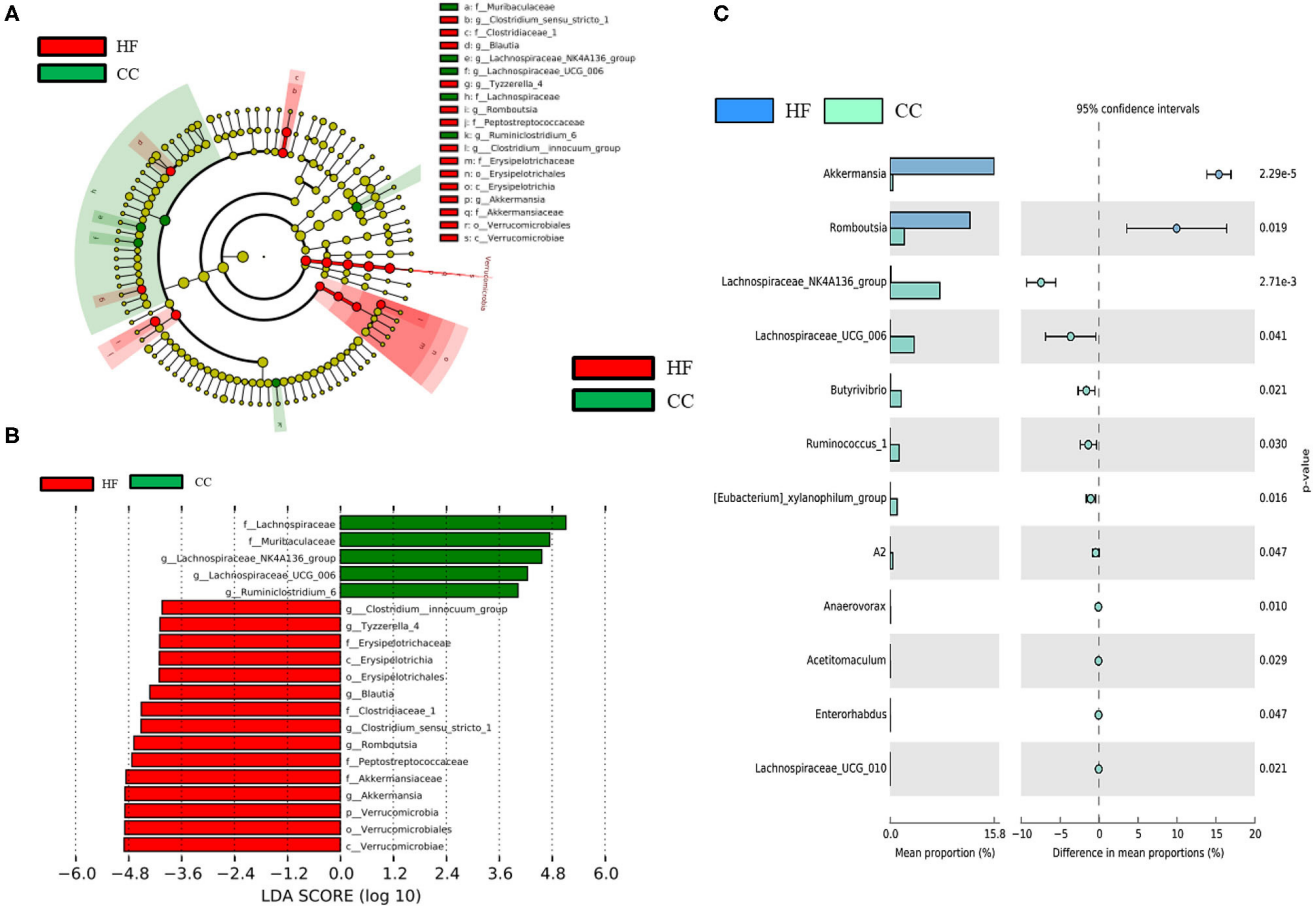
LEfSe analysis identified crucial bacteria associated with exposure to a high-fat diet (HF) in rats. **(A)** Cladogram representing taxa at all phylogenetic levels indicated predominant bacteria associated with a control diet (CC) and HF diet. For the cladogram section, the circle from the inside to the outside indicates the classification stratum from the gate to the genus. Each small circle indicates a classification at that stratum, and the diameter of the circle represents its relative abundance. The colors indicate significant differences in the biomarkers and grouping between the two groups. **(B)** For the LDA distribution histogram, taxa that reached a linear discriminant analysis score (log_10_) of >2.0 were highlighted and labeled. **(C)** At the genus level, a significant difference was observed between the CC and HF groups. (*n* = 6 for each group).

Although the F/B ratio increased in the HF group in our study, this increase was not statistically significant. Subsequently, the taxa abundance of the HF and CC dams was determined through linear discriminant analysis (LDA) and effect size (LEfSe) analysis (Figure 7). LEfSe analysis is performed to identify differences in flora and types of microorganisms between groups, which aids the development of biomarkers (17). The size of the different species is represented by the length of the histogram (i.e., LDA score) (28). The histogram revealed that HF dams (red) had more of the *Romboutsia* genus and *Akkermansia* genus than did the CC dams (green). However, the CC dams had more of the *Lachospiraceae* genus than did the HF dams. The genera that were increased in the HF group belonged to the Firmicutes phylum (Figure 7C). The predicted functions of different gut microbiomes between CC and HF groups indicated a relationship with amino acids, glucose, and lipid metabolism (Supplementary Figure 1).

### HF Diet Intake Changes Plasma SCFAs and the Gene Expression of G-Protein-Coupled Receptor 43 in Placenta

**Figure 8:**
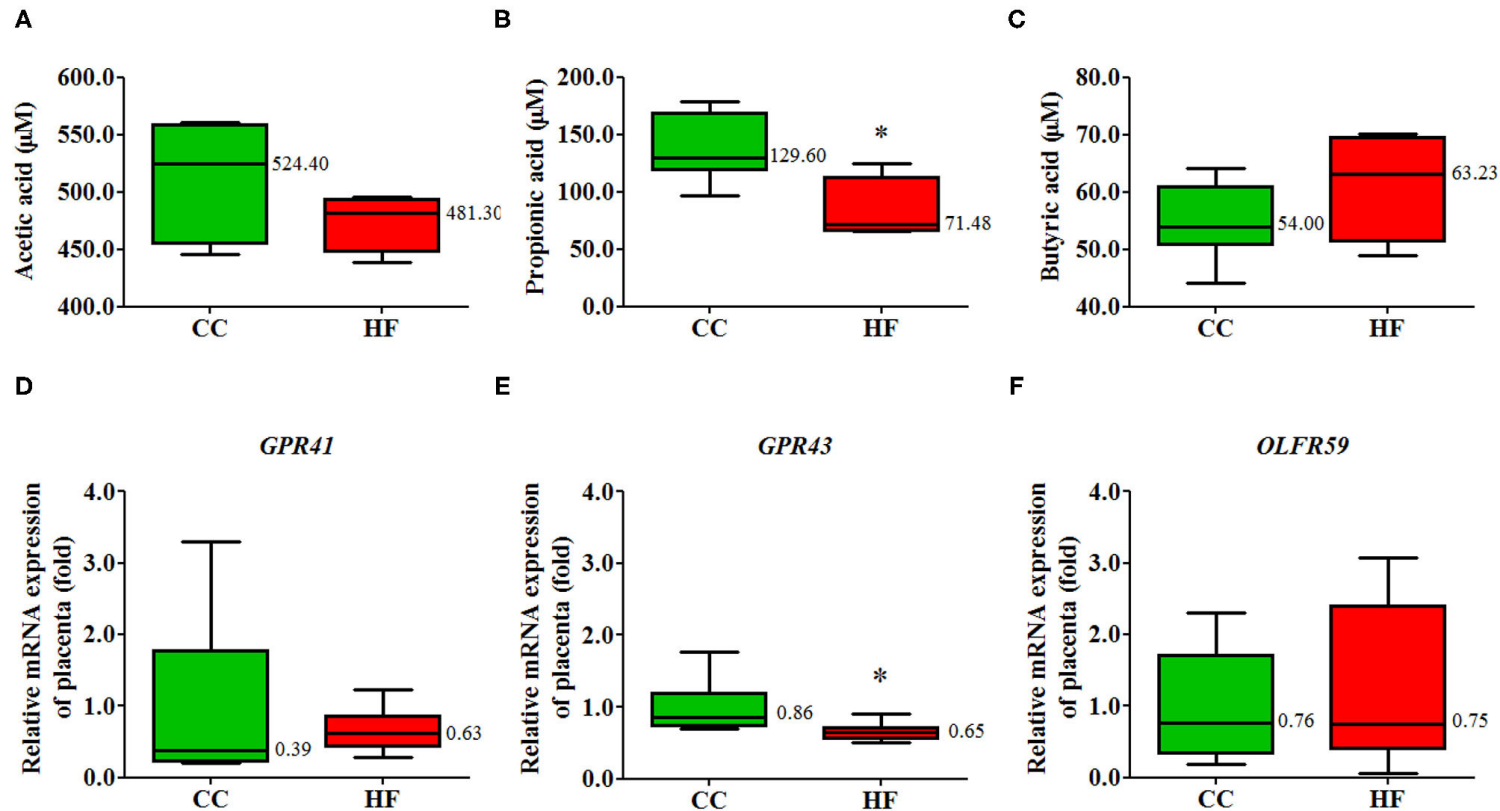
Plasma short-chain fatty acid (SCFA) levels and gene expression of corresponding receptors in placenta tissue. Plasma **(A)** acetic acid, **(B)** propionic acid, and **(C)** butyric acid levels of the rats with high-fat (HF) diet or control (CC) diet determined using gas chromatography. The mRNA expression of **(D)** *GPR41*, **(E)** *GPR43*, **(F)** *OLFR59* in placental tissue. The housekeeping gene was 18S ribosomal RNA, and mRNA expression is presented in terms of relative fold (*n* = 6 for each group). *P < 0.05. The median was shown. GPR, G-protein-coupled receptor; OLFR59, olfactory receptor 59.

Considering that dysbiosis is associated with an HF diet, we hypothesized that SCFAs would be affected in the HF group. The plasma propionate level was significantly lower in the HF group than in the CC group; however, the levels of acetate and butyrate were similar in the two groups (Figure 8A). In addition to the decreased plasma propionate level in the HF group, we found that the corresponding mRNA expression of G-protein-coupled receptor (GPR) 43, an SCFA receptor, in the placenta decreased due to a maternal HF diet, whereas the mRNA expression levels of GPR41 and OLFR59 were not affected (Figure 8B).

### Maternal HF Diet Programs Fetal Liver Steatosis Through Alteration of the key Enzyme in Lipid Metabolism

**Figure 9:**
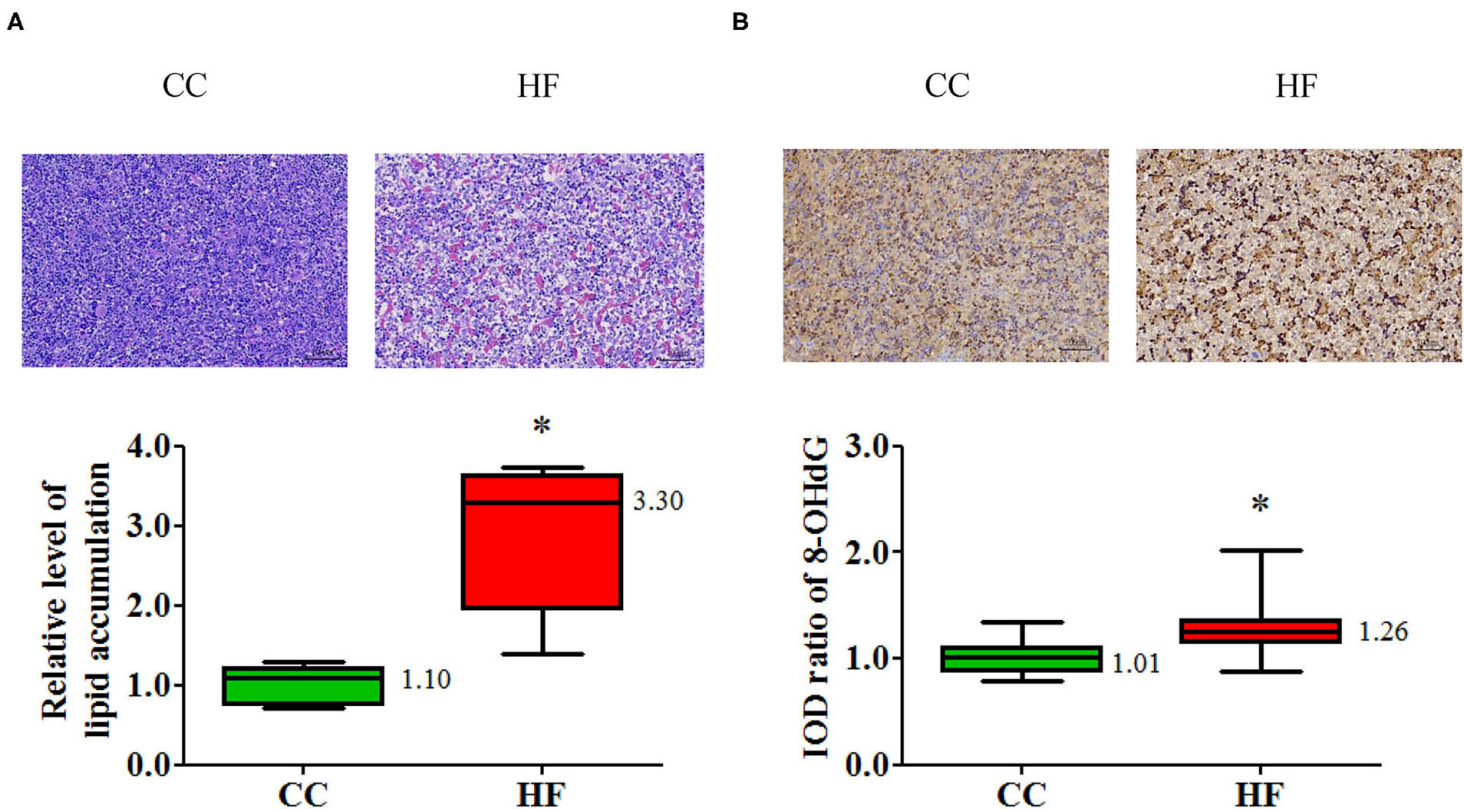
Histological changes in fetal liver associated with a maternal high-fat (HF) diet. **(A)** Degree of fetal hepatic steatosis increased with a maternal HF diet and manifested as increased vacuolation in H&E staining. **(B)** Fetal liver oxidative stress was higher with a maternal HF diet compared with a maternal control diet (CC). Oxidative stress was determined based on 8-hydroxy-2-deoxyguanosine (8-OHdG). *P < 0.05. (*n* = 6 for each group) The median was shown.

**Figure 10:**
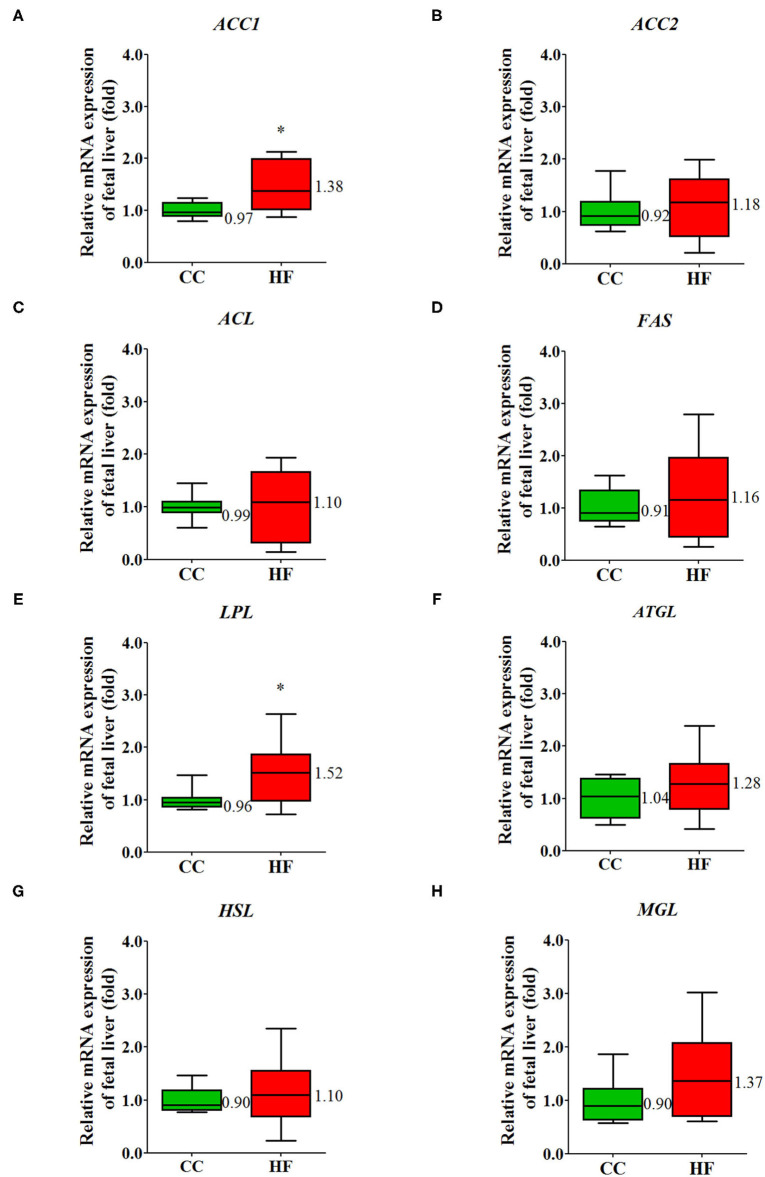
Maternal high-fat diet disrupts the key enzymes in the lipid metabolism of fetal liver. The mRNA expression of target gene. **(A)** *ACC1*, **(B)** *ACC2*, **(C)** *ACL*, **(D)** *FAS*, **(E)** *LPL*, **(F)** *ATGL*, **(G)** *HSL*, **(H)** *MGL*. The housekeeping gene was GAPDH, and mRNA expression is presented in terms of relative fold. *P < 0.05 (*n* = 8 for each group). The median was shown. ACC1, acetyl-CoA carboxylase 1; ACC2, acetyl-CoA carboxylases 2; ACL, ATP-citrate synthase; FAS, fatty acid synthase; LPL, lipoprotein lipase; ATGL, adipose triglyceride lipase; HSL, hormone-sensitive lipase; MGL, monoacylglycerol lipase.

Increasing evidence indicates that non-alcoholic fatty liver disease may begin at birth or even *in utero* and may continue on to adulthood (29). The pathway of non-alcoholic fatty liver disease (NAFLD) in placental transcriptome changed significantly due to obesity and a maternal HF diet (Table 1). To investigate the manifestation of fetal fatty liver with maternal HF diet and the mechanism through which this developmental priming is mediated, fetal livers were sampled for further study. In a histological examination, fetal livers exhibited extensive fat deposition in the HF group relative to the CC group; this was indicated by an increased proportion of vacuolation in H&E staining (Figure 9A). Furthermore, the oxidative stress determined by 8-OHdG increased in the fetal livers when mothers were fed an HF diet (Figure 9B). To uncover the programming mechanism, we analyzed the fetal liver mRNA expression of key enzymes corresponding to lipid metabolism. The genes for coding the key enzyme involved in lipid metabolism, such as acetyl-CoA carboxylase (ACC) 1 and LPL, were significantly affected by maternal HF diet (Figure 10).

## Discussion

According to previous studies, pregnant women who are obese or who have an HF diet are more likely to have offspring with obesity or metabolic problems (20, 30–32). To explore the fetal programming mechanism, placenta adaptation and changes in the maternal gut microbiome were examined in this study. We found that maternal HF diet/obesity can lead to placental remodeling through labyrinth zone hypoplasia, oxidative stress increases, GPR43 expression decreases, and alterations in metabolism-related transcriptomes. Furthermore, a maternal HF diet altered the maternal gut microbiome and decreased serum propionate. Placental remodeling and maternal dysbiosis elicit imbalances in key enzymes in lipid metabolism, oxidative stress, and steatosis in the fetal liver (graphic abstract).

The placenta is plastic and can adapt to the maternal environment to optimize the growth and development of the fetus. Studies have demonstrated that the total cortisol level of pregnant women with obesity during pregnancy is lower than that of pregnant women (33). In addition, the placental 11β-HSD-2 barrier of obese pregnant women is also upregulated, which further reduces the glucocorticoid exposure of the fetus of obese pregnant women without obesity (34). However, the effect of obesity on the metabolism of placental glucocorticoids has not been well determined. Placental dysfunction and damage are closely related to embryonic and fetal damage (8). The labyrinth zone is the layer nearest to the fetus and is composed of maternal sinusoids, trophoblastic septa, and fetal capillaries. The proportion of the placenta constituted by the labyrinth zone increases with gestational time (8). The labyrinth zone plays a role in gaseous exchange, nutrient provision, and waste removal for the fetus. Syncytiotrophoblasts in the labyrinth zone constitute a barrier that separates fetal circulation from maternal circulation. These cells have several outflow and inflow transporters that regulate chemical transfer between the mother and fetus (8). Compared with other regions of the placenta, the labyrinth zone is more susceptible to toxicological damage because of its high blood flow, active cell proliferation, and long proliferation period (8). Labyrinth zone damage is related to intrauterine growth restriction (35). The labyrinth zone is a hormone-dependent tissue, and estrogen is an inhibitor of placental growth. Estrogen overproduction or drugs with estrogenic effects may induce labyrinth zone hypotrophy (36, 37). Furthermore, labyrinth zone hypotrophy can be caused by other factors, such as the presence of immunosuppressants (e.g., glucocorticoid, azathioprine, and cisplatin) or maternal undernutrition (8). Both a maternal HF diet and undernutrition can influence the labyrinth zone; thus, the labyrinth zone tissue of the placenta is also susceptible to nutrition-related effects.

Differences in the expression of some placental genes between the CC and HF groups were identified through NGS and validated through reverse transcription PCR. Spermidine can induce autophagy and exhibits antiaging effects in multicellular organisms, including nematodes, flies, and mice. Spermidine provides several beneficial effects with caloric restriction, which partially protects against cardiovascular problems and cancers in rodent models (38). Spermidine synthetase (SRM) catalyzes spermidine production from putrescine and decarboxylated S-adenosylmethionine. High-nutrient diets upregulate the gene expression of SRM in the placenta and fetal liver of mini-pigs (39). Therefore, increased SRM in the placenta of dams with HF diet exposure is a compensatory effect that must be further investigated. The mu class of glutathione S-transferase functions to detoxicate electrophilic compounds, including some carcinogens, environmental toxins, and products of oxidative stress, through conjugation with glutathione (40). Elongation of long-chain fatty acid family member 6 (Elovl6) is an enzyme that functions in the elongation of saturated and monounsaturated fatty acids with 12, 14, and 16 carbon atoms. Elovl6 was reported to play a crucial role in lipid metabolism and insulin sensitivity. Polyunsaturated fatty acids in the diet can suppress Elovl6 expression (41). Mice with targeted disruption in the gene for Elovl6 (Elovl6 –/–) are resistant to diet-induced insulin resistance (42). Huang et al. demonstrated that Actg2, Cnfn, Muc16, and Serpina3k are at the gene network core in the placental tissue and that the genes Tkt, Acss2, and Elovl6 served as the network core in the gonadal fat tissue for mice with an HF diet (43). In our study, Elovl6 gene expression was greatly repressed in the placenta of the rats with an HF diet. Thus, Elovl6 may play a crucial role in fetus priming with a maternal HF diet. Other altered KEGG pathways in the placenta transcriptomes under a maternal HF diet/obesity include oxidative phosphorylation, arginine and proline metabolism, and NAFLD. The functional pathways indicated by the gut microbiome of the dams under an HF diet and obesity are fatty acid metabolism, fatty acid elongation in the mitochondria, arginine and proline metabolism, and biosynthesis of unsaturated fatty acids (Supplementary Figure 1). Thus, a maternal HF diet/obesity for dams can remodel the placenta and shape the gut microbiome to lipid dysmetabolism.

Fetal liver was selected for further study because the placnetal transcriptoms of the dams under the HF diet/obesity exhibited the NAFLD pathway. The fetal liver exhibited changes in the fatty liver and an increase in oxidative stress. In humans, the LPL gene expression and LPL activity of liver were higher in obese patients than in controls (44). Increased LPL activity could enhance the ability of hepatocytes to capture circulating triglycerides, leading to steatosis typically being observed in these patients. In mammals, glucose is converted into citrate in the mitochondria. Citrate is transported into the cytosol and cleaved into acetyl-CoA and oxaloacetate by ATP citrate lyase. ACC then carboxylates acetyl-CoA into malonyl-CoA. The FAS agglomerates malonyl-CoA and acetyl-CoA into a long-chain fatty acid (45). In our study, we observed a higher gene expression of LPL and ACC isoform 1 in fetal liver associated with maternal HF diet/obesity. Because disrupted hepatic metabolism and steatosis occur before any differences in body weight or body composition are observed (46), the dysregulation of hepatic lipid metabolism may be responsible for the programming of subsequent metabolic diseases in offspring with maternal HF diet/obesity.

Increased 8-OHdG levels in the placenta and fetal liver under a maternal HF diet suggest increased oxidative stress in both organs (47). Increased placental oxidative stress in maternal obesity is considered to lead to poor neonatal outcomes (48). The upregulation of nicotinamide adenine dinucleotide phosphate oxidase 2, a major source of reactive oxygen species, was suggested to be responsible for oxidative stress (49). Furthermore, oxidative damage marker significantly increased in the offspring liver with maternal HF diet/obesity. Reduced levels of glutathione peroxidase-1, the enzyme involved in antioxidation, was suggested to cause fatty liver in the offspring of mothers with obesity (46).

Among SCFAs, acetate, propionate, and butyrate are the most abundant (≥95%), with an approximate molar ratio of 3:1:1 (18, 50). Most SCFAs are produced through gut microbial anaerobic fermentation, and only a small portion is absorbed directly from food. Thus, SCFAs are produced depending on diet, microbiome constitution, and residence time in the intestinal tract (7, 8). Accumulating evidence indicates that SCFAs are key to the maintenance of health and crucial in disease development, including with regard to intestinal integrity, inflammation presentation, immune modulation, and metabolic homeostasis (18). Regarding the FFAR, both GPR41 and GPR43 are closely related to metabolic processes and have become potential targets for the treatment of type 2 diabetes, cardiovascular disease, and metabolic syndrome (19). GPR43 can stimulate insulin secretion and inhibit the apoptosis of islets cells (51). Moreover, GPR43 modulates the peptide YY–glucagon-like peptide-1 pathway for the intestine to achieve metabolic homeostasis (52). Increased expression levels of GPR41 and GPR43 in fetal membranes and placenta were noted after labor onset. Through GPR43, propionate can reduce the LPS-induced neutrophil chemotaxis and IL-8 secretion of amnion explants (53). In our study, we found that both serum propionate level and placental GPR43 expression decreased in the HF group. Maternal obesity or HF diet is usually associated with placental inflammation, which presents as an increase in proinflammatory cytokine abundance and macrophage accumulation (54, 55). Thus, the change in the propionate–GPR43 axis could be a factor that heightens inflammation associated with an HF diet.

Another study reported that pregnant C57BL/6J dams have a higher proportion of Clostridium and Akkermansia but a lower proportion of Lachnospira and Ruminococcus genera when an HF diet is adopted (13). Our results partly agree with this finding. Furthermore, an abundance of Verrucomicrobia (from phylum to order) was noted in our study. Although a lower proportion of Lachnospira and Ruminococcus genera was suggested to cause hypo-butyrate in dams owing to their butyrate-producing ability (13), this was not the case in our study. Our HF diet/obesity dams exhibited lower plasma propionate levels but similar butyrate levels compared with the control dams. Owing to complex interactions among different genera, the production of SCFAs may not be completely explained by the relative abundance of a few genera alone.

On the basis of the tail-cuff measurement, the HF diet administered before mating and during pregnancy resulted in higher systolic BP than did the chaw diet. Our results are consistent with those of several reports that indicating that an HF diet during pregnancy causes higher BP in animals on the basis of tail-cuff measurements (56–58). By contrast, one study reported no difference in mean artery pressure, measured using a carotid catheter, between rats receiving an HF diet and those receiveing a control diet during pregnancy (59). This inconsistency in the results was partially explained by the difference in measurement methods.

One limitation of this study was maternal obesity being induced by an HF diet. Whether the gut microbiomes were similar to those in other obesity models, such as high-fructose or high-glucose models, was unknown. One study examined the changes in the gut microbiome of rats with obseity induced by an HF + high-fructose (HFF) diet and an HF + high-sucrose (HFS) diet. At the species level, a significant increase in Limosilactobacillus reuteri and Bacteroides fragilis in the HFF group and an increase in Brachycybe producta in the HFS group were observed (60). Thus, other obesity models may have different gut mocribiome profiles.

## Conclusions

These findings jointly suggest that maternal HF diet or obesity shapes the composition of the maternal gut microbiota and remodels placenta, resulting in placental oxidative stress increase and lipometabolism disruption in fetal liver, which may ultimately contribute to the programming of offspring obesity.

## Data Availability Statement

The datasets presented in this study can be found in online repositories. The names of the repository/repositories and accession number(s) can be found below: NCBI SRA; PRJNA746978.

## Ethics Statement

The animal study was reviewed and approved by the Experimental Animal, the Institutional Animal Care, and Use Committee of Chang Gung Memorial Hospital (Approval Number: 2019053001).

## Author Contributions

Y-WW, H-RY, J-MS, M-MT, Y-LT, and L-TH contributed to design the work. H-RY, C-CC, I-CL, and Y-JL contributed to data acquisition. H-RY, C-CT, Y-JL, K-AC, and L-TH performed data analysis and interpretation. Y-WW, H-RY, JMS, M-MT, Y-JL, and C-CT drafted the manuscript. Y-WW, H-RY, Y-JL, C-CT, K-AC, and L-TH finalized the article. All authors have read and approved the final manuscript and agreed to be accountable for all aspects of the work.

## Funding

This research was funded in part by grants CMRPG8J0871, CMRPG8J0872, CMRPG8I0291 (H-RY), and CMRPG8H1301 (C-CT) from Chang Gung Memorial Hospital, and MOST 109-2314-B-182-040 (H-RY) from the Ministry of Science and Technology, Taiwan.

## Conflict of Interest

The authors declare that the research was conducted in the absence of any commercial or financial relationships that could be construed as a potential conflict of interest.

## Publisher's Note

All claims expressed in this article are solely those of the authors and do not necessarily represent those of their affiliated organizations, or those of the publisher, the editors and the reviewers. Any product that may be evaluated in this article, or claim that may be made by its manufacturer, is not guaranteed or endorsed by the publisher.
