# Supplementary material for: Maternal Obesity Related to High Fat Diet Induces Placenta Remodeling and Gut Microbiome Shaping That Are Responsible for Fetal Liver Lipid Dysmetabolism
Source: Front Nutr. 2021 Dec 15;8:736944. doi: 10.3389/fnut.2021.736944 (PMC8715080; doi:10.3389/fnut.2021.736944)
Supplement: Supplementary file 1 [file Table_1.DOCX]

Supplementary Table 1. Primer sequences used for quantitative real-time PCR analysis

| *Fgf23*  ([fibroblast growth factor 23](https://en.wikipedia.org/wiki/Fibroblast_growth_factor_23)) | Forward ACGGAACACCCCATCAGACTATC |
| --- | --- |
|  | Reverse TATCACTACGGAGCCAGCATCC |
| *Pdia5*  (protein disulfide isomerase family A Member 5) | Forward ATATGACCGAGCTGTGACGCTGAA |
|  | Reverse ACATCTTTGGCTCCAGGGTCTTCT |
| *Map2k3*  (mitogen activated protein kinase kinase 3) | Forward CCATTCTGCGATTCCCTTAC |
|  | Reverse GCAATGTCCGTCTTCTTAGT |
| *Prl7b1*  (prolactin-7B1) | Forward AACAATGCCTCTGGCCACTGC |
|  | Reverse AGGCCATTGATGTGCTGAGACAGT |
| *Ndst1*  (N-deacetylase and N-sulfotransferase 1) | Forward GATGACCCGGTGGCCCTAAA |
|  | Reverse TCTGTTCGCAGCAGTTTGCC |
| *Lfng*  (LFNG *O*-fucosylpeptide 3-beta-N-acetylglucosaminyltransferase) | Forward TTCATCGCCGTCAAGACCAC |
|  | Reverse GTGCTTGGCCAAAGCTTCATC |
| *Rrad*  (Ras related glycolysis inhibitor and calcium channel regulator) | Forward AGGGACAGCAAAGAGGATAATG |
|  | Reverse TTGGGCTTAGAGAACAGAGAGG |
| *Prl2b1*  (prolactin family 2, subfamily b, member 1) | Forward TGTCATCCTTGCAGTCAAGC |
|  | Reverse GGCAGCGAATCAGGGTATAA |
| *Orm1*  (orosomucoid 1) | Forward GTGTTCAAGCAGGCAGTTCAAA |
|  | Reverse TCTGTGGTCTGAAACTCCCGA |
| *Smoc1*  (SPARC related modular calcium binding 1) | Forward ACCACCGGCCCCAGGTTTCTAAT |
|  | Reverse CTACCTCGATGGACCACGGCC |
| *GPR41*  (G-protein-coupled receptor 41) | Forward TGACGGTGAGCATAGAACGTTT |
|  | Reverse GCCGGGTTTTGTACCACAGT |
| *GPR43*  (G-protein-coupled receptor 43) | Forward CACCGAGAACCAAATCACCT |
|  | Reverse GAGGGACTCTGCCTCAAGTG |
| *OLFR59*  (olfactory receptor 59) | Forward TGGTTTGGCTTCCCCCTACT |
|  | Reverse AGGTGGACAAAGCCAGATCAA |
| *ACC1*  (acetyl-CoA carboxylase 1) | Forward TGAGGAGGACCGCATTTATC |
|  | Reverse GCATGGAATGGCAGTAAGGT |
| *ACC2*  (acetyl-CoA carboxylase 2) | Forward CGCTGCGGTCAAGTGT |
|  | Reverse CGTTGGCGTAGTTGTTATT |
| *ACL*  (ATP citrate lyase) | Forward ACCCAGAGGAAGCCTACATTGC |
|  | Reverse TTCGCCAGTTCGTTGACACC |
| FAS  (fatty acid synthetase) | Forward AGATCCTGGAACGTGAACATGA |
|  | Reverse GCCGTACTTCACGAATGGGT |
| LPL  (lipoprotein lipase) | Forward GTACAGTCTTGGAGCCCATGC |
|  | Reverse GCCAGTAATTCTATTGACCTTCTTGTT |
| *ATGL*  (adipose triglyceride lipase) | Forward TGTGGCCTCATTCCTCCTAC |
|  | Reverse TCGTGGATGTTGGTGGAGCT |
| *HSL*  (hormone sensitive lipase) | Forward GCTGGGCTGTCAAGCACTGT |
|  | Reverse GTAACTGGGTAGGCTGCCAT |
| *MGL*  (monoacylglycerol lipase) | Forward TGACCAACTCTGTCCTCCAT |
|  | Reverse GCACTGCCCTTCCTCTT |
| GAPDH  (glyceraldehyde 3-phosphate dehydrogenase) | Forward TCTTGTGCAGTGCCAGCCTC |
|  | Reverse GTCACAAGAGAAGGCAGCCCTGG |
| 18S ribosomal RNA | Forward GCGATGCGGCGGCGTTAT |
|  | Reverse AGACTTTGGTTTCCCGGAAGC |

Supplementary Table 2. Placenta and offspring outcome in HF and CC dams.

|  | CC | HF | *P* value |
| --- | --- | --- | --- |
| Litter number | 13.00±0.86 | 13.17±0.83 | NS |
| Litter gender (M/F) | 1.63±0.42 | 0.85±0.24 | NS |
| Litter size (g) | 2.41±0.23 | 3.34±0.49 | NS |
| Placenta weight (g) | 0.39±0.01 | 0.40±0.02 | NS |

Supplementary Table 3. The 26 upregulated genes in the placenta of the HF group compared with those of the CC group.

| Gene description (Up gene) | Gene name | FPKM | | Log2 ratio |
| --- | --- | --- | --- | --- |
|  |  | HF value | CC value | HF/CC |
| spermidine synthase [Source:RGD Symbol;Acc:620796] | Srm | 0.91 | 0.00 | 16.47 |
| glutathione S-transferase mu 3 [Source:RGD Symbol;Acc:11402080] | NEWGENE_620381 | 0.67 | 0.00 | 16.04 |
| ubiquitin specific peptidase 9, Y-linked [Source:RGD Symbol;Acc:9087771] | Usp9y | 0.43 | 0.00 | 15.39 |
| SPARC/osteonectin, cwcv and kazal like domains proteoglycan 3 [Source:RGD Symbol;Acc:1305672] | Spock3 | 0.36 | 0.00 | 15.14 |
| similar to 40S ribosomal protein S2 [Source:RGD Symbol;Acc:1562399] | RGD1562399 | 46.92 | 4.99 | 3.23 |
| tubulin, alpha 1C [Source:RGD Symbol;Acc:1307226] | Tuba1c | 8.35 | 1.01 | 3.05 |
| breast cancer metastasis-suppressor 1 homolog [Source:RGD Symbol;Acc:11501278] | LOC108348098 | 7.22 | 0.93 | 2.96 |
| similar to heat shock protein 8 [Source:RGD Symbol;Acc:1595502] | LOC680121 | 31.89 | 4.64 | 2.78 |
| hemoglobin subunit epsilon 1 [Source:RGD Symbol;Acc:1305115] | Hbe1 | 114.48 | 16.66 | 2.78 |
| atrophin-1-like [Source:RGD Symbol;Acc:6491795] | LOC100911672 | 2.01 | 0.31 | 2.70 |
| LFNG O-fucosylpeptide 3-beta-N-acetylglucosaminyltransferase [Source:RGD Symbol;Acc:620587] | Lfng | 8.94 | 1.40 | 2.68 |
| serine/threonine-protein kinase PAK 2-like [Source:RGD Symbol;Acc:6497075] | LOC100910732 | 12.06 | 1.99 | 2.60 |
| heterogeneous nuclear ribonucleoprotein H3 [Source:RGD Symbol;Acc:1310019] | Hnrnph3 | 26.81 | 4.67 | 2.52 |
| - | Rn60_1_2212.4 | 8860.32 | 1550.23 | 2.51 |
| RRAD, Ras related glycolysis inhibitor and calcium channel regulator [Source:RGD Symbol;Acc:69357] | Rrad | 17.93 | 3.46 | 2.37 |
| heterogeneous nuclear ribonucleoprotein A/B [Source:RGD Symbol;Acc:9343685] | LOC103689931 | 16.92 | 3.46 | 2.29 |
| Prolactin family 2, subfamily b, member 1 [Source:RGD Symbol;Acc:620118] | Prl2b1 | 328.06 | 67.77 | 2.28 |
| similar to heat shock protein 8 [Source:RGD Symbol;Acc:1586445] | LOC688655 | 11.78 | 2.43 | 2.28 |
| orosomucoid 1 [Source:RGD Symbol;Acc:67390] | Orm1 | 93.74 | 20.86 | 2.17 |
| 60S ribosomal protein L13-like [Source:RGD Symbol;Acc:2318188] | LOC100362400 | 39.69 | 9.70 | 2.03 |
| glyceraldehyde-3-phosphate dehydrogenase [Source:RGD Symbol;Acc:11369796] | LOC108351137 | 71.82 | 18.06 | 1.99 |
| high mobility group nucleosome binding domain 5B [Source:RGD Symbol;Acc:1308736] | Hmgn5b | 30.26 | 8.40 | 1.85 |
| hemoglobin subunit beta-2-like [Source:RGD Symbol;Acc:9285450] | LOC103694855 | 700.35 | 195.59 | 1.84 |
| doublesex- and mab-3-related transcription factor C1 [Source:RGD Symbol;Acc:11451771] | LOC108348153 | 50.59 | 14.51 | 1.80 |
| SPARC related modular calcium binding 1 [Source:RGD Symbol;Acc:1303126] | Smoc1 | 30.65 | 9.94 | 1.62 |
| thymosin, beta 10-like [Source:RGD Symbol;Acc:2321025] | LOC100364435 | 610.22 | 200.90 | 1.60 |

Supplementary Table 4. The 27 downregulated genes in the placenta of the HF group compared with those of the CC group.

| Gene description (Down gene) | Gene name | FPKM | | Log2 ratio |
| --- | --- | --- | --- | --- |
|  |  | HF value | CC value | HF/CC |
| N-deacetylase and N-sulfotransferase 1 [Source:RGD Symbol;Acc:69303] | Ndst1 | 8.93 | 25.95 | -1.54 |
| ribosomal protein L30 [Source:RGD Symbol;Acc:621201] | Rpl30 | 46.11 | 162.37 | -1.82 |
| tubulin, alpha 8 [Source:RGD Symbol;Acc:1566041] | Tuba8 | 16.59 | 66.14 | -2.00 |
| solute carrier family 6 member 9 [Source:RGD Symbol;Acc:621243] | Slc6a9 | 10.42 | 42.29 | -2.02 |
| calcium binding and coiled coil domain 1 [Source:RGD Symbol;Acc:619812] | Calcoco1 | 2.83 | 11.71 | -2.05 |
| apolipoprotein A1 [Source:RGD Symbol;Acc:2130] | Apoa1 | 22.37 | 93.34 | -2.06 |
| prolactin family 7, subfamily b, member 1 [Source:RGD Symbol;Acc:708582] | Prl7b1 | 18.41 | 88.35 | -2.26 |
| WW domain-binding protein 11-like 1 [Source:RGD Symbol;Acc:9435381] | Wbp11l1 | 1.10 | 5.27 | -2.27 |
| apolipoprotein B [Source:RGD Symbol;Acc:2129] | Apob | 0.74 | 3.62 | -2.30 |
| stress-70 protein, mitochondrial-like [Source:RGD Symbol;Acc:6486342] | LOC100912578 | 3.64 | 20.11 | -2.47 |
| alpha-fetoprotein [Source:RGD Symbol;Acc:2065] | Afp | 38.68 | 238.46 | -2.62 |
| paraneoplastic Ma antigen family member 5 [Source:RGD Symbol;Acc:1565857] | Pnma5 | 3.45 | 26.35 | -2.93 |
| mitogen activated protein kinase kinase 3 [Source:RGD Symbol;Acc:1306620] | Map2k3 | 0.99 | 8.26 | -3.06 |
| protein disulfide isomerase family A, member 5 [Source:RGD Symbol;Acc:1359236] | Pdia5 | 4.39 | 37.07 | -3.08 |
| serine/threonine-protein phosphatase 2A catalytic subunit alpha-like [Source:RGD Symbol;Acc:2318135] | LOC100362453 | 1.47 | 18.13 | -3.63 |
| mitochondrial fission factor [Source:RGD Symbol;Acc:1310230] | Mff | 0.75 | 9.57 | -3.66 |
| protein TFG-like [Source:RGD Symbol;Acc:9131838] | LOC103690095 | 0.53 | 7.32 | -3.78 |
| patatin-like phospholipase domain containing 2 [Source:RGD Symbol;Acc:1309044] | Pnpla2 | 0.75 | 10.68 | -3.84 |
| rhox homeobox family member 2-like [Source:RGD Symbol;Acc:11409898] | LOC108348152 | 2.48 | 36.83 | -3.89 |
| carboxypeptidase B2 [Source:RGD Symbol;Acc:71035] | Cpb2 | 0.00 | 0.44 | -15.42 |
| - | AABR07042903.2 | 0.00 | 0.47 | -15.52 |
| transgelin 3 [Source:RGD Symbol;Acc:69301] | Tagln3 | 0.00 | 0.51 | -15.64 |
| fibroblast growth factor 23 [Source:RGD Symbol;Acc:620178] | Fgf23 | 0.00 | 0.59 | -15.84 |
| fucose mutarotase-like [Source:RGD Symbol;Acc:6494411] | LOC100911225 | 0.00 | 0.76 | -16.22 |
| - | Rn50_1_0101.1 | 0.00 | 0.82 | -16.32 |
| ELOVL fatty acid elongase 6 [Source:RGD Symbol;Acc:620585] | Elovl6 | 0.00 | 1.03 | -16.65 |
| - | AABR07066510.1 | 0.00 | 3.15 | -18.26 |
