# Supplementary figures and images for: Maternal Obesity Related to High Fat Diet Induces Placenta Remodeling and Gut Microbiome Shaping That Are Responsible for Fetal Liver Lipid Dysmetabolism
Source: Front Nutr. 2021 Dec 15;8:736944. doi: 10.3389/fnut.2021.736944 (PMC8715080; doi:10.3389/fnut.2021.736944)

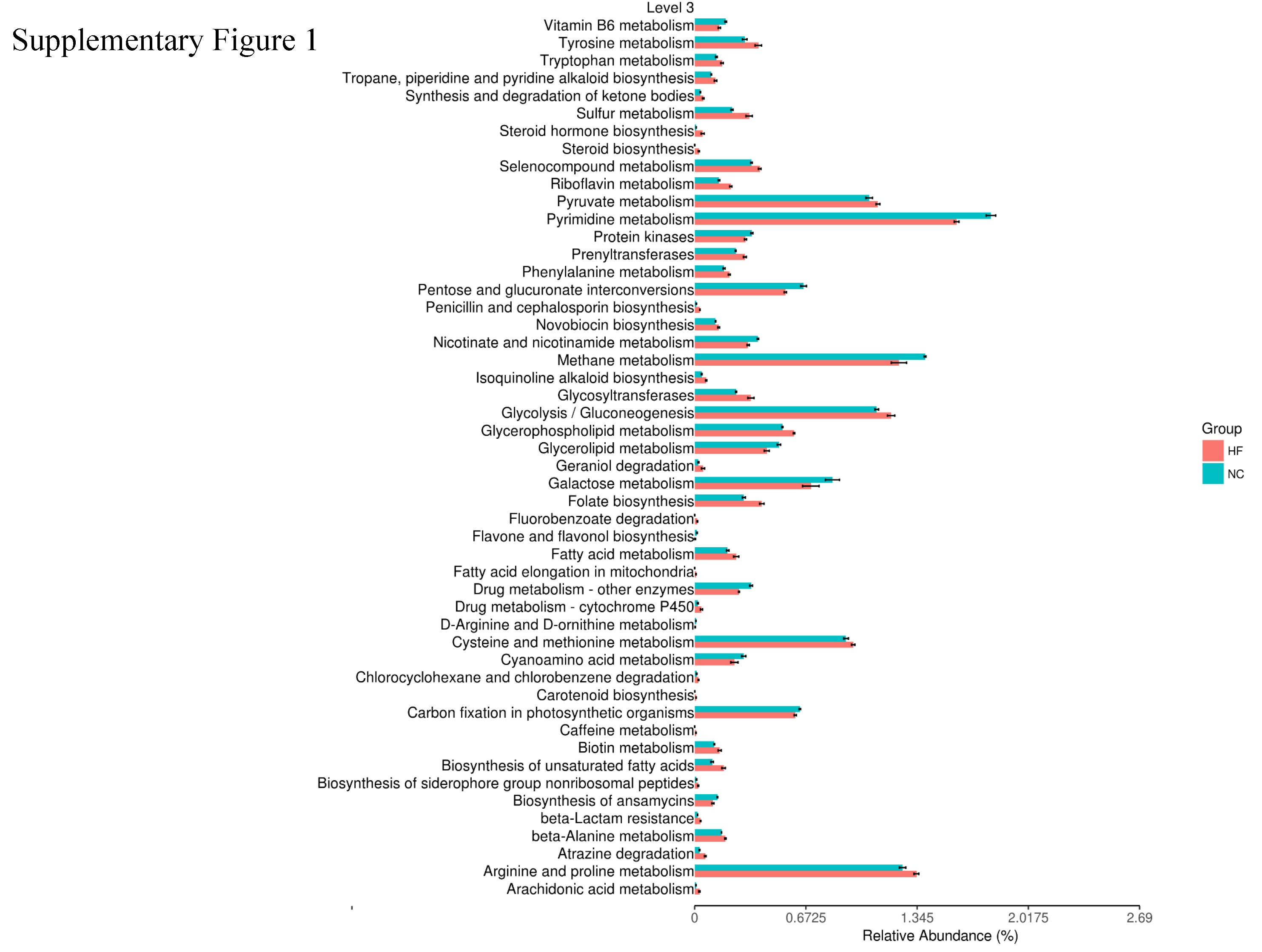

Supplement: Supplementary file 2 [file Image_1.TIFF]
